# Epidemiological behavior of the COVID-19 contamination curve in Brazil: Time-series analysis

**DOI:** 10.1371/journal.pone.0268169

**Published:** 2022-09-22

**Authors:** Thiffany Nayara Bento de Morais, Ketyllem Tayanne da Silva Costa, Gustavo Nepomuceno Capistrano, Fábia Barbosa de Andrade

**Affiliations:** Department of Nursing, Federal University of Rio Grande do Norte, Rio Grande do Norte, Brazil; The University of the West Indies, TRINIDAD AND TOBAGO

## Abstract

Brazil is experiencing the greatest episode of sanitary collapse ever known in the country’s history. Therefore, the relevance of this study is highlighted for the scientific advance of the epidemiological behavior of the virus in Brazil, enabling the development of analyses and discussions on the factors that influenced the high rates of contamination by SARS-CoV-2 in the country. Given the above, this study aims to analyze the epidemiological behavior of the COVID-19 contamination curve by epidemiological weeks (EW), in the years 2020–2021, in Brazil. This is an ecological study of time series, prepared using information collected through secondary means. The country of origin of the study is Brazil, and its main theme is the number of people infected during the COVID-19 pandemic, this being the dependent variable of the study. The data has been analyzed from February 23, 2020, when the first case was confirmed in Brazil, to January 1, 2022. In 2021, the country’s graph shows an exponential growth, reaching a peak of approximately 250 new cases per 100,000 inhabitants in the 12th EW. This data represents the highest rate of the pandemic in Brazil, and did not vary significantly for the next twelve weeks. Thus, it was identified that Brazil was severely impacted by the new coronavirus, considering the high rates of confirmed cases of the virus in the country, the low adherence of the population to preventive measures, the late start of mass vaccination in the Brazilian population, and the lack of structure in the health system, which was not appropriately prepared for the high demand generated by COVID-19.

## Introduction

SARS-CoV-2, the virus that causes COVID-19, was first reported in late December 2019 in Wuhan, Hubei Province, China, but soon spread to other countries. In addition to the rapid spread, the virulence of the new coronavirus led the World Health Organization (WHO) to declare the outbreak a public health emergency of international concern and subsequently a pandemic on March 11, 2020 [1,2].

COVID-19 mainly causes respiratory symptoms, which can progress to pneumonia, and it also leads to other systemic complications, such as cardiovascular, metabolic, and renal complications. In addition, many patients require hospitalization and, in some cases, intensive care [3].

COVID-19 has already spread throughout the world, impacting nearly 190 countries, with the highest incidence across America, Europe, and Southeast Asia. According to the data from the dashboard on COVID-19, developed and managed by Johns Hopkins University (2022), as of February 22, 2022, the new coronavirus had infected 427,358,056 people and led to the death of 5,904,723 people, worldwide [4].

The country that presents the highest numbers for COVID-19 is the United States of America, with approximately 78.6 million people infected and 939 thousand deaths from the disease. Next are India—with about 42.8 million infected and 512 thousand deaths—and Brazil, with 28.3 million infected—less than those in India, but a higher number of deaths—approximately 645 thousand [4]. This characteristic of the rapid expansion of the virus highlights the need for active participation and loco-regional and international collaboration for the effective control of the high transmission rate and in the field of prevention and treatment [5].

To minimize the effects of the virus, health authorities, together with governments, have instituted preventive measures, which mainly include the use of masks, hand hygiene, and social distancing, considering that the primary means of transmission of the virus is through droplets and aerosols expelled through the respiratory tract. In cases of a positive test for the new coronavirus, it is necessary that the infected persons isolate themselves, and the epidemiological investigation of their close contacts is conducted [6].

According to the Oswaldo Cruz Foundation (2021), Brazil is experiencing the greatest episode of sanitary collapse ever known in the country’s history [7]. Therefore, the relevance of this study is highlighted for the scientific advance on the epidemiological behavior of the virus in Brazil, enabling the development of analyses and discussions on the factors that influenced the high rates of contamination by SARS-CoV-2 in the country.

Given the above, the study aimed to analyze the epidemiological behavior of the COVID-19 contamination curve by epidemiological weeks, in the years 2020 and 2021, in Brazil.

## Materials and methods

This research is an ecological study of time series, prepared using information collected through secondary means. The country of origin of the study is Brazil, and its main theme is the number of people infected during the COVID-19 pandemic, this being the dependent variable of the study. The data from February 23, 2020, when the first case was confirmed in Brazil, to January 1, 2022, was analyzed. The standardization of the period occurred by epidemiological weeks (EW), from the 9th EW of 2020 to the 53rd EW of 2021. The territory was analyzed considering the regions of Brazil, which show different dates of confirmation of the first case of infection.

The data was collected on January 19, 2022, from a website designed by the Brazilian Ministry of Health to disseminate the COVID-19 information, which is updated daily and can be easily accessed via <https://covid.saude.gov.br/>.

For a better reading of the dependent variable, the morbidity rate due to SARS-COV-2 was calculated, in which the number of infected people was divided by the number of inhabitants and then multiplied by 100,000 (1). Furthermore, the independent variable for the research was the epidemiological weeks.


$$\text{M}=\left(\text{Morbidity}\;\text{by}\;\text{COVID}-19\right)/\text{Population}\;\text{X}\;100,000$$ (1)

The variables were divided considering the relevance and dissemination of COVID-19, thus, the outcome and choice as dependent variable the records of illnesses. The choice of independent variables focused mainly on time, taking the occurrence of the event in each week, or in other words, the use of the Epidemiological Week.

As for the reality of the BIAS the study had data variation in each week, there were no outliers. To maintain the quality of the statistical treatment, the Joinpoint system made the correction using the linear regression technique.

The inclusion criteria consisted of people who became ill with COVID-19 only in all age groups within a time interval from February 23, 2020 to January 1, 2022. As for the exclusion criteria, all other respiratory diseases or other influenza syndromes not classified with a final diagnosis of COVID-19 were excluded from the database in the filtering process.

The next step after data collection was to clean the data in Microsoft Excel® software and then perform a more rigorous analysis in Joinpoint software, version 4.9.0.0 (Surveillance Research, National Cancer Institute, USA), provided by the National Cancer Institute of the United States (http://surveillance.cancer.gov/joinpoint/) open to all public with free access.

Joinpoint, through linear regression, elaborates junction points in the temporal series. Thus, two outputs are formed: the Annual Percentage Change (APC) and the Average Annual Percentage Change (AAPC), which are responsible for informing the significant variations in the analyzed rate. The Monte Carlo Permutation model was used to demonstrate the significance in the tests. Finally, the demonstration of the results was done through epidemiological curves, presenting a better view of the spread of COVID-19 in Brazil from 2020 to 2021.

The information obtained for the purpose of this study was of secondary character and was obtained through databases that have public domains; hence, it was not necessary to use personal data, and appreciation of the Research Ethics Committee was not required.

## Results

Tables 1 and 2 demonstrate the synthesis of data achieved through linear regression of COVID-19 cases in Brazil organized by epidemiological weeks (EW), from 2020 and 2021, respectively. Thus, Table 1 shows the presence of four Joinpoints in Brazil and most of its regions, except Midwest that had five. In addition, Brazil presents all APCs with statistical relevance, totaling five, including the Southeast, but the other regions have four. Meanwhile, from the AAPC perspective, the Midwest region presents the most inconstancy in the numbers of infected while the Southeast presents the most constancy.

**Table 1:** COVID-19 morbidity rate in Brazil and its regions in 2020. Brazil, 2022.

Local	Joinpoint^1^	Period	WPC^2^	Lower	Upper	AWPC^3^	Lower	Upper
Brazil	22, 31, 45, 50	9 to 22	48.1*	38.9	57.9	13.3*	10.9	15.8
		22 to 31	7.8*	5.3	10.3			
		31 to 45	-6.0*	-7.0	-4.9			
		45 to 50	19.3*	11.6	27.5			
		50 to 53	-9.6*	-17.8	-0.5			
North	19, 22, 34, 42	12 to 19	94.3*	57.6	139.6	13.3*	8.8	18.0
		19 to 22	43.9*	9.1	89.8			
		22 to 34	-1.8*	-3.2	-0.3			
		34 to 42	-8.3*	-11.7	-4.9			
		42 to 53	2.7	0.6	4.8			
Northeast	22, 30, 45, 50	10 to 22	52.5*	40.5	65.6	11.9*	8.8	15.0
		22 to 30	4.6*	1.1	8.3			
		30 to 45	-8.0*	-9.3	-6.7			
		45 to 50	21.6*	9.3	35.3			
		50 to 53	-10.2	-22.1	3.5			
Midwest	25, 31, 38, 45, 50	10 to 25	52.3*	42.6	62.6	15.8*	12.6	19.1
		25 to 31	13.0*	7.2	19.0			
		31 to 38	-1.9	-5.3	1.6			
		38 to 45	-12.4*	-16.1	-8.4			
		45 to 50	10.4*	0.7	20.9			
		50 to 53	-5.6	-17.5	8.0			
Southeast	22, 31, 45, 49	9 to 22	38.9*	30.7	47.6	11.8*	9.3	14.3
		22 to 31	9.1*	6.3	12.0			
		31 to 45	-6.2*	-7.4	-5.0			
		45 to 49	21.7*	7.7	37.4			
		49 to 53	-1.1	-7.4	5.6			
South	28, 36, 41, 51	11 to 28	33.0*	27.4	38.9	13.9*	11.1	16.7
		28 to 36	7.3*	3.2	11.6			
		36 to 41	-15.7*	-23.6	-7.1			
		41 to 51	17.0*	14.2	19.8			
		51 to 53	-28.9*	-43.0	-11.4			

^1^by epidemiological week.

^2^week percentage change.

^3^average week percentage change.

*p-value <0.05.

**Table 2:** COVID-19 morbidity rate in Brazil and its regions in 2021. Brazil, 2022.

Local	Joinpoint^1^	Period	WPC^2^	Lower	Upper	AWPC^3^	Lower	Upper
Brazil	12,24	1 to 12	3.4*	1.2	5.7	-4.1	-5.0	-3.3
		12 to 24	-0.3	-2.4	1.8			
		24 to 52	-8.5*	-9.4	-7.6			
North	3, 7, 11, 25, 39	1 to 3				-2.5*	-4.3	-0.7
			24.9	-1.3	58.1			
		3 to 7	-8.7	-18.1	1.7			
		7 to 11	6.8	-4.4	19.4			
		11 to 25	-4.6*	5.8	-3.4			
		25 to 39	-11.4*	-13.5	-9.3			
		39 to 52	5.6*	2.4	8.9			
Northeast	11, 25, 32	1 to 11	6.0*	3.5	8.6	-3.3*	-4.6	-2.0
		11 to 25	1.2	-0.1*	2.6			
		25 to 32	-22.0*	-27.0	-16.7			
		32 to 52	-3.6*	-5.5	-1.6			
Midwest	7, 10, 34	1 to 7	-1.8	-9.1	6.2	-3.4	-5.9	-0.9
		7 to 10	22.3	-17.1	80.6			
		10 to 34	-2.2*	-3.0	-1.3			
		34 to 52	-9.2*	-11.5	-6.9			
Southeast	25	1 to 25	1.3*	0.1	2.6	-4.3*	-5.5	-3.1
		25 to 52	-9.0*	-10,9	-7.1			
South	5, 10, 14, 25	5 to 10	25.3	7.9	45.5	-4.7	-7.2	-2.1
		10 to 14	-17.8	-34.1	2.4			
		14 to 25	2.7	-1.0	6.6			
		25 to 52	-8.6*	-9.9	-7.2			

^1^by epidemiological week.

^2^week percentage change.

^3^average week percentage change.

*p-value <0.05.

The linear regression of 2021 is demonstrated in Table 2. Brazil presented two Joinpoints, the Southeast region only one, Center-West had three, South had four, and North had five, demonstrating distinct behaviors. In relation to the statistical relevance of the APC in Brazil, two were obtained, from the 1st to 12th EW and 24th to 52nd EW. The Midwest and Southeast also had two APCs, the Northeast demonstrated the highest with three, and South with only one, from the 25th to 52nd EW. When the AAPC was evaluated, the South had the most inconsistent numbers while the North had most consistent numbers, in terms of the number of people infected with COVID-19.

**Fig 1:**
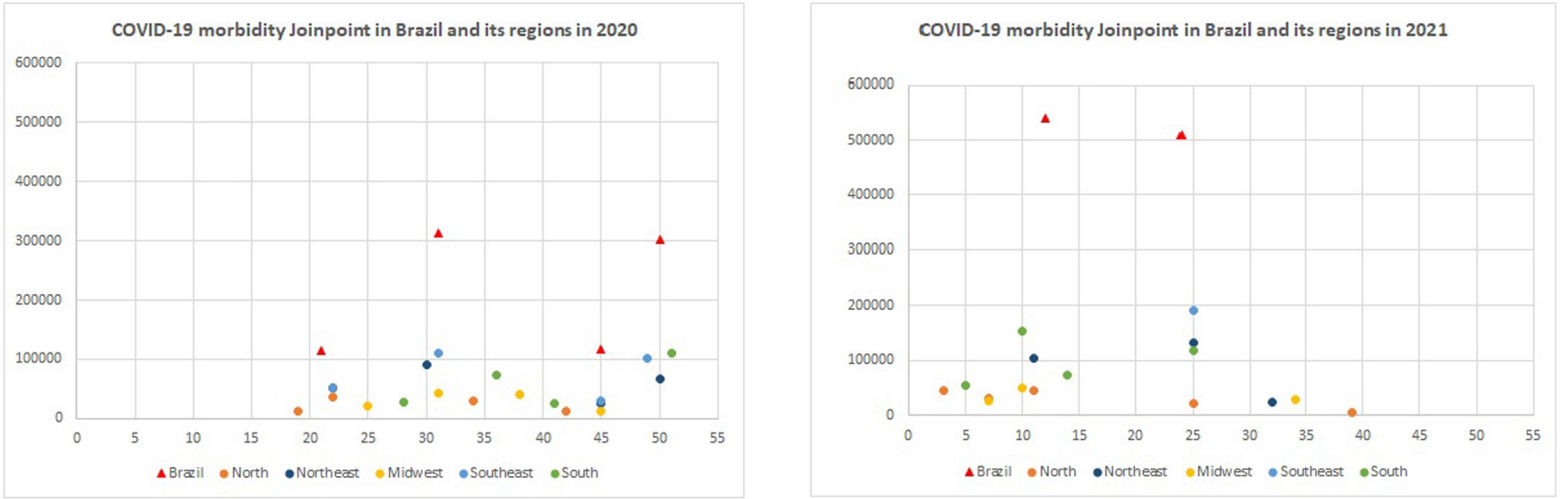
COVID-19 morbidity Joinpoint in Brazil and its regions from 2020 to 2021. Brazil, 2022.

Fig 1 represents the data shown in Tables 1 and 2, by means of a scatter plot of the Joinpoints in Brazil and in each region by EW in 2020 and 2021. In relation to Brazil, in 2020, the first Joinpoint appears after EW 20 and grows gradually, remaining in the range of 300,000 infected, with a drop at EW 45, followed by a new increase at EW 50. In 2021, the morbidity rate increased considerably, with more than 500,000 infected cases between EW 12 and 24.

Regarding the specificities of the regions, it is possible to observe in the year 2020, a greater concentration of Joinpoint between weeks 19 and 51, with the North presenting an initial and final Joinpoint earlier than the other regions, and also obtaining the lowest rates. The Southeast and South regions present higher Joinpoint and rates, but in different periods (EW 31, EW 51, respectively). Besides this, EW 45 calls attention because 3 regions had Joinpoints in this week and then presented a growth in rates and a new higher Joinpoint.

In 2021, the concentration occurred between EW 3 and 15, only presenting new Joinpoints around EW 25, in which 4 regions (North, Northeast, Southeast and South) and Brazil presented Joinpoints in the same period. The behavior of 2020 was repeated in the year 2021 in each region, with the South and Southeast being the regions with the highest morbidity rates, while the North presents the lowest numbers, although it has an earlier initial Joinpoint (EW 3) and a later final one (EW 39).

**Fig 2:**
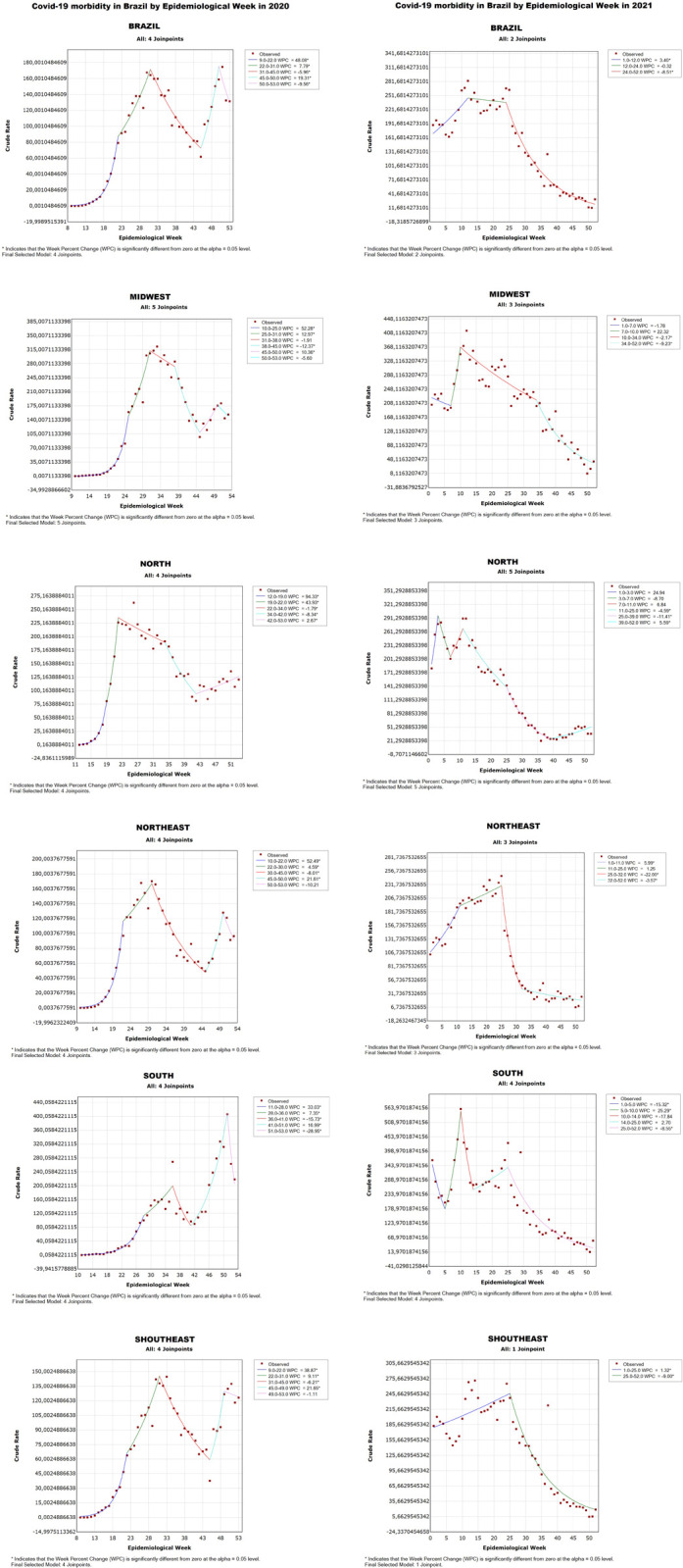
COVID-19 morbidity rate in Brazil and its regions from 2020 to 2021. Brazil, 2022.

Based on Fig 2, it is possible to observe the incidence of the number of infected persons in Brazil from 2020 to 2021 through polynomial models. Thus, a significant increase in the number of people infected by COVID-19 was seen in Brazil in 2020 until approximately the 30th EW (with a rate of 160 per 100,000 people), followed by a significant decrease in cases until the 46th EW, with approximately, 80 cases per 100,000 people. However, between the 44th and 50th EW there was an increase that reached a peak with a rate of more than 170 cases, but, a drop can be witnessed again until the 53rd EW, which ended in 2020.

In this way, the Southeast, Northeast, and Midwest regions of Brazil present an ascending curve in the graph similar to that of entire Brazil in 2020, with a representative increase in the rate until the 30th EW. As for North, there was a rise in numbers until the 24th EW, with the highest rate of the region in 2020 (225 cases per 100,000 inhabitants), followed by a subtle decrease (rate of 200 per 100,000 inhabitants in the 24th EW). The North showed another decrease in infected cases until the 42nd EW, with approximately 100 cases per 100,000 inhabitants. Thereafter, the rate increased to 125 cases per 100,000 inhabitants in 51st EW.

The illness has varied over time and this shows that in Brazil the behavior of the epidemiologic transition respected the reality of ascension in the linear regression panel, but, there was a statistical variability between some Brazilian regions, which reveals the epidemiologic profile of the COVID-19 aggravations. The Brazilian population has shown points of oscillation among the regions with falling results observed in the North, Northeast, Midwest, South, and Southeast. It is worth noting that there are realities of greater population contribution in some regions, as well as a greater amount of health services implemented in certain Brazilian states that integrate the regions. Thus, the Southeast, South and Midwest regions have more financial resources. However, there is a different reality in the North and Northeast regions that have less funding and, consequently, less installed capacity. This regional difference brings a distinct epidemiological profile in Brazilian illness.

Meanwhile, the South presented the peak in the number of cases in the 53rd EW, reaching the highest rate with more than 400 cases per 100,000 inhabitants. Still, the drop between the 32nd and 46th EW in Brazil was similar to that of the Northeast, Southeast, and Midwest. A similar pattern occurred again, with all regions showing an increase in the number of cases at the end of 2020.

From the perspective of the year 2021, Fig 2 indicates that the first 12 EWs of the year show an increase in the graph, reaching approximately the rate of 251 cases per 100,000 inhabitants, and this value stabilizes in the following weeks, more specifically, until the 23rd EW. However, this pattern changes with a gradual and representative drop in the number of infected cases and continues until the end of the year, which has the lowest rate with 12 cases per 100,000 inhabitants.

The Northeast and Southeast regions show similar behavior, as cases in the beginning of 2021 increased until the 25th EW with a rate of 231 and 245, respectively, followed by an accentuated decrease with a rate of approximately 7 in both regions in the 52nd EW. The Midwest started the year with a different behavior, as there was a decrease in the number of infected cases between the 0th and 7th EW with a rate of 208, followed by a considerable increase, demonstrated by a steep upward curve over the course of three weeks, reaching the region’s peak in the year 2021 with a rate of 368. However, the numbers gradually dropped, once again, until the 52nd EW of 2021.

Another region that shows an analogous pattern at the beginning of 2021 was the South, as the cases began with a decrease until the 5th EW with 178 cases per 100,000 inhabitants, followed by an accentuated progression, reaching the peak with a rate of 563 in the 10th EW. However, the numbers dropped sharply until the 13th EW with a rate of just over 233 infected cases. This was followed by a subtle increase, (rate of 343 in the 25th EW), followed by a gradual decline that lasted until the end of the year 2021.

In contrast, the North, much like Brazil and the South, began the year 2021 with an increase in the rate until approximately the 3rd EW, demonstrating its peak with a rate of 291 cases. It was followed by a drop (rate of 201 in the 7th EW) and a subsequent increase (rate of 261 in the 12th EW). Thereafter the behavior was analogous to the other regions, with a considerable drop in numbers until the final EWs of the year 2021. COVID-19 started to be confirmed in Brazil in the 8th EW of 2020, particularly in the southeast region, while other regions tested positive for the virus in the subsequent weeks, with the northern region presenting the most delayed confirmed cases (11th EW).

With this, the graph of Brazil, presented in Fig 2, shows a gradual increase until the 15th EW, with less than one case per 100,000 inhabitants. This was followed by a period of rapid increase in the number of cases until the 30th EW, recording more than 160 cases per 100,000 inhabitants. Thereafter, the graph shows a drastic decline in the rate, with confirmation of approximately 70 new cases per 100,000 inhabitants. Finally, the year ended with a rise that peaked in morbidity at more than 170 proportional cases, followed by a drop of about 20 cases per 100,000 inhabitants in the 2020 rate.

In 2021, the country’s graph shows an exponential increase, reaching a peak of approximately 250 new cases per 100,000 inhabitants in the 12th EW; the highest rate of the pandemic in Brazil which did not vary significantly for the next twelve weeks. It was followed by a gradual decline in the rate of confirmed cases, which reached about 20 infected people per 100,000 inhabitants by the end of the year.

In this sense, the quick dissemination of the virus caused a collapse in the world health system, especially in underdeveloped countries like Brazil. Therefore, during the pandemic, particularly during the morbidity and mortality peaks of COVID-19, the lack of supplies, beds, equipment, and skilled labor, became a common problem worldwide.

## Discussion

In the year 2020, health professionals and researchers in the field faced a challenging scenario due to the high rate of COVID-19 infection, considering that this was a recent disease and with incipient studies on transmissibility pattern, infectivity, lethality, and mortality, without specific and effective vaccines and drugs to treat the virus [8].

Knowing that this virus spreads mainly by droplets and aerosols expelled by the respiratory tract, measures to prevent and contain the virus were adopted, such as hand sanitation; social distancing; cancellation of events and closure of public and private institutions; allowing operation of only essential activities, such as hospitals, pharmacies and supermarkets; and recommended quarantine of the infected persons and their close contacts. [9]. The use of masks was not recommended at that time, as there were not enough studies proving their effectiveness in preventing the coronavirus [10].

The main public affected by the COVID-19 infection is the elderly person, which can be associated with an impaired immune system, which becomes less effective with the aging process, inflammation and exacerbated amount of cytokines, as well as epigenetic changes and underlying comorbidities [11].

The activities in the field of health also underwent changes, with partial and restricted functioning. The health services primarily focused on coronavirus cases, which led to the reallocation of professionals from other sectors to the COVID-19 units, to alleviate the high demand generated by the crisis. [12]. With this, many patients with chronic diseases and who were on continuous treatment were negatively affected due to the reduction in specialized care.

There were abrupt changes in social dynamics, with the rapid advance of the virus causing high morbidity and mortality rates. Moreover, the scarcity of scientific evidence and, concomitantly, an excess of often contradictory theories and information caused confusion and uncertainty in the population [13].

All these factors contributed to the increasing rate of infection of the virus in Brazil, which is evident in Fig 2, that shows an ascending curve until EW 30, reaching the peak of the period with more than 160 cases per 100,000 inhabitants. Different regions of the country presented an epidemiological behavior similar to the overall pattern observed in Brazil, with the exception of the Northern region, which saw an increase of up to 225 cases per 100,000 inhabitants in EW 22, and the Southern region, where cases increased until EW 36, reaching the peak of the period with a rate of around 200 per 100,000 inhabitants.

Following this period of increased infection, the country experienced a reduction of confirmed cases of COVID-19 from EW 30 to 44, which may be related to the consolidation of more scientifically based studies on effective methods of prevention, as well as the structuring and greater investments in health, such as the construction of field hospitals, increasing the amount of free diagnostic tests for the population, and the incentives to adopt preventive measures [14].

Another assertive action to combat the high rate of transmissibility is to recognize the region and analyze what the main measures should be taken in relation to the particular demands of each location and better coordination in policy and financial plan, as the research points out that these actions were very successful in the Caribbean [15].

Besides public health issues, the economy has also been greatly affected by the pandemic, with increased economic vulnerability, especially in middle-income countries [16].

Another important factor in curbing the spread of the virus was the economic incentive of R$ 600.00, established by the Brazilian government through Decree No. 10.316, dated April 7, 2020, with the objective of supporting families in situations of social vulnerability, who survived on informal jobs that caused a greater exposure to the virus [17].

Even after the decline in the epidemic curve, the country faced a new wave of infections, which led to the peak of contamination in the year 2020, in EW 49, with a rate of approximately 180 cases per 100,000 inhabitants (Fig 2).

This increase may have been influenced by the election campaign period, starting in September and ending on voting day, on November 15, 2020, corresponding with the EW 47, as allowed by Resolution no. 23,627, of August 13, 2020 [18]. During this period, there were several cases of agglomeration throughout the Brazilian territory, often without the adherence to preventive measures, such as the use of masks and frequent use of alcohol gel, which may reflect the increase in cases.

Due to all these factors, the WHO declared South America as the epicenter of COVID-19, and Brazil became one of the most affected countries, assuming the second position in the morbidity ranking in the year 2020, after the United States of America [19,20]. Towards the end of 2020, the pattern in some regions showed reduction in the rate of infection, with the exception of the North, which continued to see an increase in the infection rate until the year end.

With the increase in morbidity, the population began to be more committed to social isolation. This adherence remained low throughout the pandemic in Brazil, reaching 52.3% in December 2020, the highest percentage since May 2020. A decrease in the number of confirmed cases could be observed in the last epidemiological weeks of the year. However, this was not a lasting change, as the isolation rate gradually decreased in the following year, reaching 31.1% in February 2021, reflecting the new increase in the COVID-19 morbidity rate [21].

In the same period in 2021, the epidemiological curve in Brazil, presented a jump in the first 12 EWs, reaching the rate of approximately 251 cases per 100,000 inhabitants. Even the South, which started the year with a low rate of infection, saw the peak of the pandemic in EW 10, with a rate of about 563 cases per 100,000 inhabitants. This may have been influenced by the Carnival, an event of Brazilian popular culture, which comprises crowded events. Even though it was forbidden due to the pandemic, there were many clandestine agglomerations in EW 7.

At that time, COVID-19 vaccines were already available in the market, with scientific proof of efficacy. However, due to high demand and issues related to commercial transactions between countries and the vaccine manufacturing companies, Brazil started vaccination late as compared to other countries in Europe and North America [22].

Between EW 12 and 23, the curve was stable at the peak, and thereafter showed a drop from EW 23, which corresponds to the month of June, until the end of the year 2021. According to Our World in Data COVID vaccination data, Brazil began vaccinations against COVID-19 in the second half of January 2021, as did India. However, the US had already started vaccination in December 2020. Moreover, the speed and vaccination coverage in the US was higher, reaching the peak of daily vaccination in April 2021 with 1.06% of the total population. However, Brazil only reached the highest number of vaccinations in 24 hours, in August 2021 with 0.91%, but was already showing high rates of vaccination since June 2021 [23].

As shown in Fig 2, Brazil, as well as all of its regions showed a pattern of gradual decline of COVID-19 infections in EW 23 until the end of 2021. In the last EW, the country showed a rate of confirmed cases as approximately 11 cases per 100,000 inhabitants and, in the same period, the vaccination percentage of the first dose was 75.6% and for the complete vaccination scheme was 67.2%. These data corroborate the effectiveness of the vaccines [24].

Even with the vaccine available, a considerable group refuses to be vaccinated and spreads false information that has a negative impact on population adherence. In this regard, an Indian study found that 88% of respondents reported hesitating the vaccine due to post-vaccination adverse effects. Also, the same study observed that people with a profile of respect for social distancing recommendations in public places were more likely to be vaccinated [25].

A study in Germany interviewed 2029 participants (75.3% women) and found that only 57.5% were willing to be vaccinated, with the female public being more resistant to vaccination, however, the same public had a greater adherence to other preventive measures, such as hand hygiene and use of a face mask [26].

## Conclusion

This study performed an analysis of the epidemiological behavior of the new coronavirus contamination curve by epidemiological weeks, in the years 2020 and 2021, in Brazil. Thus, it was identified that Brazil was severely impacted by the new coronavirus, considering the high rates of confirmed cases of the virus in the country, the low adherence of the population to preventive measures, the late start of mass vaccination among Brazilians, and the lack of structure in the health system, which was not appropriately prepared for the high demand generated by COVID-19.

As for the limitations of the study, the authors searched several information systems in Brazil, but they always found impediments to carrying out the study, such as corrupted spreadsheets and the file size exceeding the software limit, which made it impossible to open the files, or even the data was not open to the public. Thus, the scarcity of variables in the public domain and difficulty of access were barriers to a better analysis of the epidemiological profile of confirmed cases of COVID-19.
